# The role of EBV-encoded miRNA in EBV-associated gastric cancer

**DOI:** 10.3389/fonc.2023.1204030

**Published:** 2023-06-14

**Authors:** Ting Liu, Xiaoying Zhou, Zhe Zhang, Yutao Qin, Rensheng Wang, Yanning Qin, Yuqi Huang, Yingxi Mo, Tingting Huang

**Affiliations:** ^1^ Department of Radiotherapy, First Affiliated Hospital of Guangxi Medical University, Nanning, China; ^2^ Guangxi Key Laboratory of High-Incidence-Tumor Prevention and Treatment, Ministry of Education, Guangxi Medical University, Nanning, China; ^3^ Department of Otolaryngology-Head and Neck Surgery, First Affiliated Hospital of Guangxi Medical University, Nanning, China; ^4^ School of Basic Medical Sciences, Guangxi Medical University, Nanning, China; ^5^ Department of Research, Guangxi Medical University Cancer Hospital, Nanning, China

**Keywords:** Epstein-Barr virus, gastric cancer, MicroRNAs, carcinogenesis, clinical application

## Abstract

Epstein-Barr virus (*human herpesvirus* 4, EBV) is a linear double-stranded DNA virus that infects over 90% of the population worldwide. However, our understanding of EBV’s contribution to tumorigenesis of EBV-associated GC (EBVaGC) remains incomplete. Recent advancements in EBVaGC research have highlighted that EBV-encoded microRNAs (miRNAs) play prominent roles in critical cellular processes such as migration, cell cycle, apoptosis, cell proliferation, immune response, and autophagy. Notably, the largest group of EBV-encoded miRNAs, known as BamHI-A rightward transcripts (BARTs), exhibit bidirectional effects in EBVaGC. For instance, they present both anti-apoptotic and pro-apoptotic functions and enhance chemosensitivity while also conferring resistance to 5-fluorouracil. Despite these findings, the comprehensive mechanisms through which miRNAs contribute to EBVaGC are yet to be fully elucidated. In this work, we summarize the current evidence of the roles of miRNA in EBVaGC, particularly with the application of multi-omic techniques. Additionally, we discuss the application of miRNA in EBVaGC in retrospective analyses and provide novel perspectives on the use of miRNA in EBVaGC in translational medicine.

## 1 Introduction

Epstein-Barr virus (EBV), formally named *Human gammaherpesvirus 4*, is a double-stranded DNA virus first identified by Epstein et al. in 1964 from African Burkitt’s lymphoma (1, 2). EBV infects over 90% of the population worldwide, with most infected individuals displaying no or mild symptoms and becoming lifelong carriers (3). EBV is transmitted *via* saliva, with B cells in the oropharynx and tonsils being the initial site of infection (4, 5). EBV expression is almost completely silenced upon entering a latent state, and the infection is maintained for life (6). Under certain conditions, a transition from a quiescent to an activated state occurs, initiating a lytic infection and continuing the infection cycle by infecting the next host cell. A small fraction of carriers is at risk of developing several types of human malignancies, including nasopharyngeal carcinoma (NPC), gastric cancer (GC), Burkitt’s lymphoma (BL), and Hodgkin lymphoma (HL) (3, 7). According to the latest global cancer statistics released by the World Health Organization (WHO), the proportion of gastric cancer incidence and mortality was approximately 5.6% and 7.7%, respectively (8). Nearly 9% of GC is attributed to EBV infection (1.3% - 30.9% according to geographic distribution) (9). Thus, EBVaGC is of interest to oncologists and researchers, primarily those focusing on the pathogenetic role of EBV in epithelial cancers.

Recent studies have found that miRNAs regulate migration, cell cycle, apoptosis, cell proliferation, immune response, and autophagy in EBVaGC. Besides, they have been shown to enhance the sensitivity of EBVaGC to radiotherapy, chemotherapy, and immunotherapy, and may also serve as potential prognostic indicators. However, the comprehensive mechanisms of miRNAs in EBVaGC tumorigenesis are awaited to be fully elucidated. Here, we summarize the current evidence of the roles of miRNA in EBVaGC, especially those utilizing multi-omic techniques, discuss the application of miRNA in EBVaGC in retrospective analyses, and provide novel perspectives on the use of miRNA in EBVaGC in translational medicine.

## 2 EBV and EBVaGC

There were an estimated 265 000 incidence cases of EBV-associated malignancies globally (18% of all NPC, GC, BL, and HL cases combined), resulting in over 164 000 deaths in 2017 (10). Notably, EBVaGC accounted for 42% of all cases (113 000) and 48% of deaths (78 600) (10), making it a noticeable disease burden worldwide. EBVaGC is identified as EBV-infected tumor cells by polymerase chain reaction analysis and *in situ* hybridization and characterized by lymphoepithelioma-like histology, unique clinical features, and relatively desirable prognosis compared to non-EBV-associated cases (EBVnGC) (11). EBVaGC is associated with Caucasian or Hispanic ethnicity, male gender, younger age, history of gastric surgery, and unhealthy lifestyles (such as smoking and consumption of salty foods) (12–15). *Helicobacter pylori* infection has long been considered a causative factor for GC. Its role in EBVaGC remains unclear and has been well discussed elsewhere (15). After infecting host cells, EBV selectively expresses latent genes and enters a stable latent phase (16, 17). There are four types of latent infection, termed type 0, I, II, and III latency (18, 19). EBVaGC exhibits latency infection type I or II, selectively expressing EBV-encoded RNAs (EBERS) (EBER-1 and EBER-2), EBV nuclear antigen 1 (EBNA-1), BamHI-A rightward transcripts (BARTs), BART microRNAs (BART miRNAs), and BamHI-A rightward frame 1 (BARF1), with most lacking or low expression of latent membrane proteins (LMP) 1 and LMP2B. LMP2A is expressed in approximately 40% of EBVaGC patients (20, 21). Somatic genomic abnormalities (such as PIK3CA mutations, ARID1A mutations, BCOR mutations, and amplification of JAK2, PD-L1, and PD-L2), and extreme DNA hypermethylation are intrinsic etiological factors contributing to EBVaGC pathogenesis (11, 22). Additionally, extensive hypermethylation of DNA promoters and altered expression of EBV-encoded microRNAs (miRNAs) are the most significant epigenetic features of EBVaGC (23–25).

## 3 The role of EBV-encoded miRNA in EBVaGC

**Figure 1:**
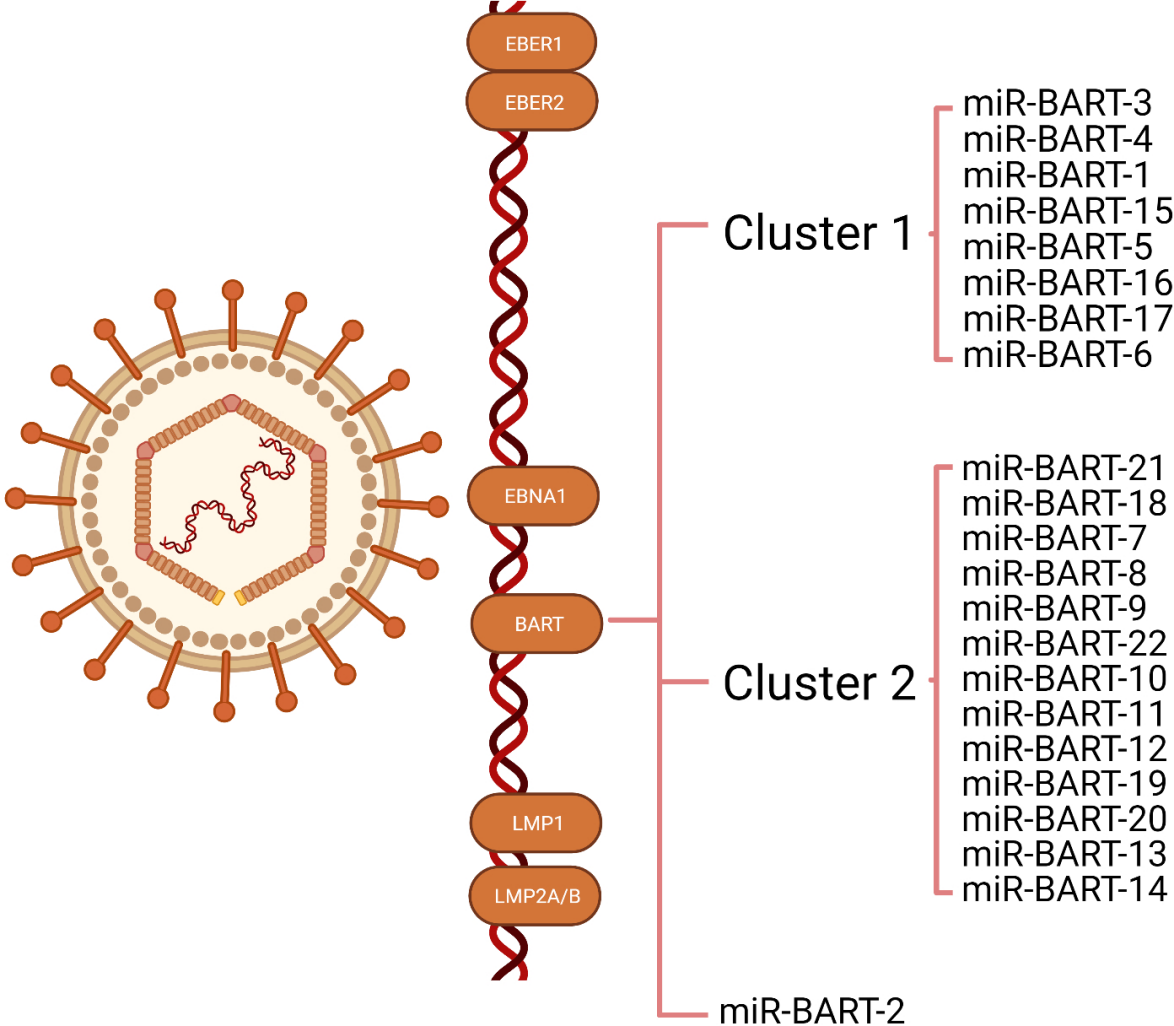
Illustration of the transcription of Epstein-Barr virus (EBV) latent genes expressed in EBV-associated gastric cancer (EBVaGC). The latency genes expressed in EBVaGC include EBV-encoded small RNA (EBER)1, EBER2, EBV nuclear antigens1(EBNA1), and BART miRNAs. Most EBVaGC tumors lack or express low levels of latent membrane proteins (LMP) 1, LMP2A, and LMP2B. The BART region contains two subclusters: 1 and 2, with miR-BART2 located downstream of these two clusters.

MiRNAs are 18-24 nucleotides short non-coding RNA (26) that can regulate their target gene expression by degrading mRNA or inhibiting protein translation. Since the first viral-encoded miRNA was identified by Pfeffer et al. in 2004, EBV has been verified to encode approximately 25 pre-miRNA and 44 mature miRNAs, which can be classified into three categories: BART-cluster1, BART-cluster2, and BHRF1-cluster (21, 27). In the BHRF1 gene, three pre-miRNA are encoded (BHRF1 to 3) that produce four mature miRNAs, and the BART region contains 22 pre-miRNA (BART1 to 22), which generate 40 mature miRNAs. Under steady-state tumor growth, EBV utilizes cellular mechanisms to generate latent gene products that sustain the latent infection (16, 17). There are four types of latent infection, termed type 0, I, II, and III latency (18, 19). EBVaGC belongs to latency infection type I or II (20, 21). The functions of BHRF in a lytic infection, cell cycle progression, virus infection, and reproduction are revealed in many tumor types, whereas they are rarely expressed in EBVaGC (28, 29). As a result, BART miRNAs control most of the functions (30, 31). There are two subclusters in the BART region: 1 and 2, with the miR-BART2 located downstream of both. The BART cluster miRNAs of EBV are highly expressed in EBVaGC (**Figure 1**).

**Figure 2:**
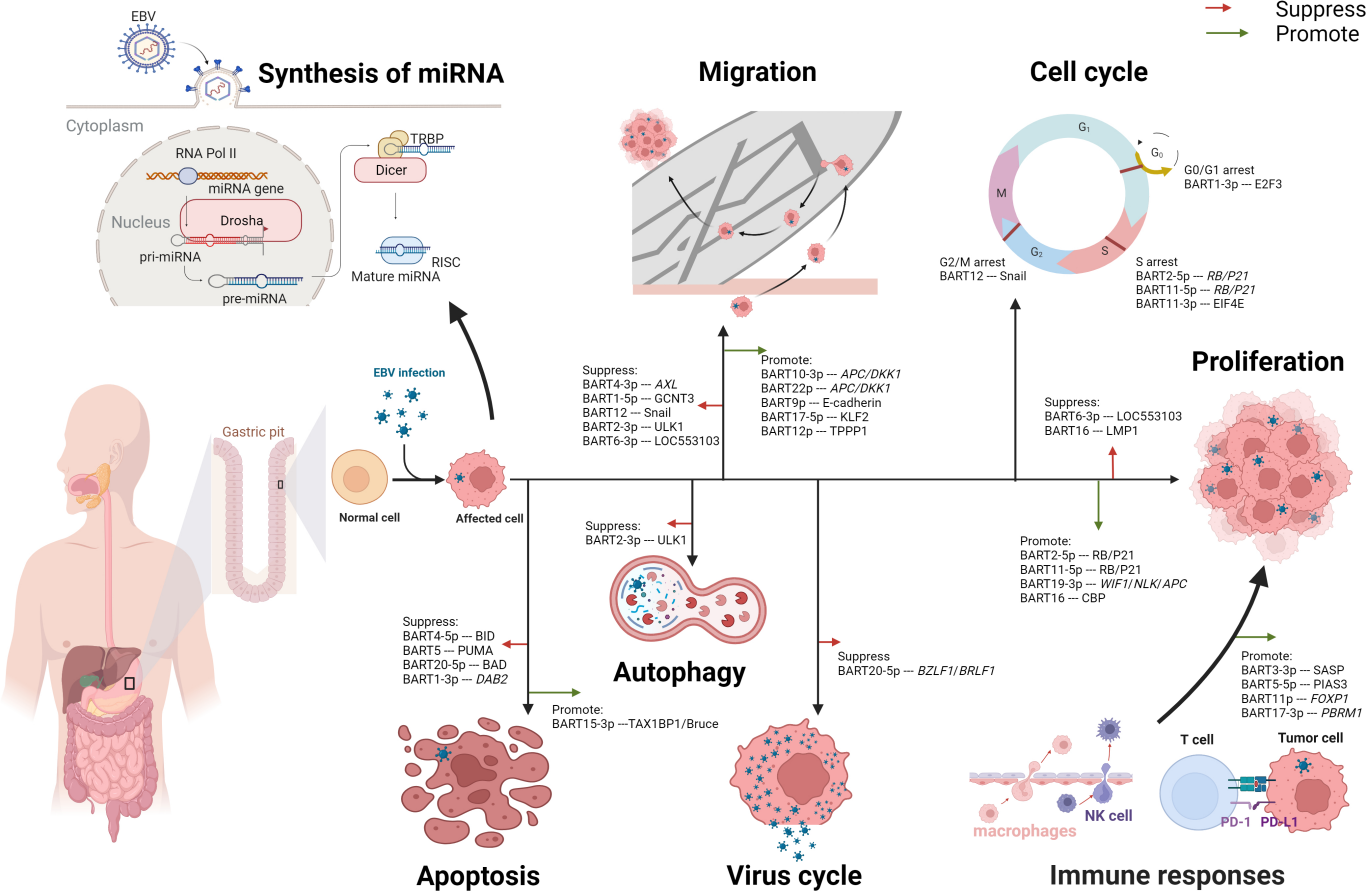
Multifaceted roles of EBV-encoded miRNAs in modulating the cell biological functions in EBVaGC. After EBV infection, the synthesis of miRNA in normal gastric cells undergoes alterations. The process begins with the transcription of EBV DNA by RNA polymerase II (RNA pol II), generating primary miRNA (pri-miRNA). Subsequently, the pri-miRNA is cleaved by the enzyme Drosha in the cell nucleus, forming precursor miRNA (pre-miRNA). The pre-miRNA is then transported to the cytoplasm, where it undergoes further cleavage by Dicer-Transactivation Response RNA-Binding Protein (TRBP), producing mature miRNA. Once mature, the miRNA associates with the RNA-induced Silencing Complex (RISC) and binds to its target mRNA, exerting inhibitory or promoting effects. These EBV-encoded miRNAs play a significant role in the oncogenesis of gastric cancer, demonstrating both suppressive and promotive effects on critical cellular processes such as migration, cell proliferation, and apoptosis. Moreover, these miRNAs can modulate the cycles of both the host cell and the virus. Notably, EBV-encoded miRNAs profoundly impact immune responses through diverse mechanisms, including the regulation of PD-L1 expression. Additionally, miRNAs function to suppress autophagy, further contributing to their multifaceted role in EBV-associated gastric cancer.

Accumulating evidence indicates that the aberrant expression of miRNA plays a crucial role in cancer development. In the following sections, we will describe the contributions of miR-BARTs in regulating migration, cell cycle, apoptosis, cell proliferation, immune response, and autophagy (**Figure 2**).

### 3.1 Migration

In EBVaGC cells, EBV-encoded miRNAs modulate epithelial-mesenchymal transition (EMT), affecting intercellular adhesion and cell motility and deformability, leading to alterations in the migration capability of the tumor cells (32). Meng-He Zhao and colleagues revealed that miR-BART4-3P targeted the *AXL* gene directly to promote the apoptosis and suppress the proliferation and migration of GC cells *via* downstream effectors of EMT, including E-cadherin, Vimentin, Zinc Finger E-box Binding Homeobox 1 (ZEB1), and phospho-AKT (P-AKT) (33). Besides, Juanjuan Liu et al. reported that miR-BART1-5p restrained GC cells proliferation and migration by targeting GCNT3 (core 2β-1, 6-acetylglucosaminyltransferase) *via* modulating the expressions of E-cadherin, N-cadherin, and Vimentin (34). MiR-BART12 targeted and accelerated the degradation of Zinc finger protein SNAI 1 (Snail), one of the EMT inducers, consequently repressing cell proliferation and migration (35). Similarly, MiR-BART2-3p was demonstrated to reduce cell migration through inhibition of Unc-51-like kinases 1 (ULK1) which may reduce the expression of β-catenin, N-cadherin, and ZEB1 (36). Moreover, Baoyu He et al. indicated that miR-BART6-3p downregulated long non-coding RNA (lncRNA) LOC553103, reversing the EMT process and suppressing the migration and invasion of cancer cells (37).

EBV-encoded miRNAs present bidirectional effects on cell migration in EBVaGC tumor cells. Min Dong and coworkers demonstrated that miR-BART10-3p and miR-BART22 targeted *adenomatous polyposis coli* (*APC*) and *dickkopf-1* (*DKK1*) to activate the Wnt signaling pathway, resulting in a downregulation in E-cadherin whereas an upregulation in Twist (38, 39). MiR-BART9 has been implicated in the modulation of E-cadherin expression, which might increase cell proliferation and invasion (40). In wound healing assays, miR-BART17-5p promoted cell migration by directly downregulating Kruppel-like factor 2 (KLF2) (41). Yingfen Wu et al. revealed that miR-BART12 bound to the 3’UTR region of Tubulin Polymerization-Promoting Protein1 (TPPP1) mRNA and downregulated TPPP1 (42), which promoted the EMT of tumor cells.

### 3.2 Cell cycle

EBV-encoded miRNAs contribute to the regulation of numerous vital factors involved in the host cell cycle, such as the E2 promoter-binding factor (E2F) family, transcriptional corepressor 1 (*RB*), and Eukaryotic translation initiation factor 4E (EIF4E) (43–45). Myung Chan Park et al. found miR-BART1-3p directly targeted the 3’UTR of E2F3 mRNA, resulting in G0/G1 arrest and suppressing cell proliferation in EBVaGC cell lines (43, 46). Besides, miR-BART2-5p and miR-BART11-5p induced S-phase arrest by targeting *RB* and *p21*, promoting cell proliferation (44, 47). Another group of researchers observed that the expression level of EIF4E in EBVaGC tissues was lower than in EBVnGC (45). Their results from the luciferase reporter assay indicated that miR-BART11-3p targeted the 3’-UTR region of EIF4E, leading to retardation at the S phase, increased apoptosis, and decreased cell migration in EBVaGC (45). Jun Li et al. suggested that miR-BART12 directly targeted Snail by interacting with its 3’-UTR, leading to G2/M phase arrest and promoting cell apoptosis (35).

On the other hand, EBV-encoded miRNAs are involved in the switch from EBV latent cycle to the EBV lytic cycle, which is usually initiated by activating the expression of two EBV immediate-early genes: *BZLF1* and *BRLF1* (48). Yu-Jin Jung et al. described that miR-BART20-5p plays a significant role in the maintenance of latency by suppressing the lytic cycle and the production of progeny virus, resulting in a lifelong infection in the host (49).

### 3.3 Apoptosis

The Bcl-2 family is one of the most important regulators of apoptosis, controlling the balance between pro-apoptotic and anti-apoptotic signals within human cells (50). Recent studies have suggested that EBV-encoded miRNAs in EBVaGC inhibit pro-apoptotic proteins in the Bcl-2 family, such as BH3 interacting domain death agonist (BID), p53-upregulated modulator of apoptosis (PUMA), and Bcl-2-associated death promoter (BAD), leading to the evasion of apoptosis (51–53). MiR-BART5, miR-BART4-5p, and miR-BART20-5p downregulated the expressions of PUMA, BID, and BAD, respectively (51–53). Kyoungmi Min et al. found that miR-BART1-3p suppressed the expression of Disabled homolog 2 (DAB2) (a tumor suppressor gene associated with apoptosis) in EBVaGC cells to evade apoptosis (54). In contrast, two studies have observed that miR-BART15-3p promoted apoptosis in EBVaGC cells (55, 56). MiR-BART15-3p restrained the expression of anti-apoptotic genes: TAX1BP1 (Tax1-binding protein 1) and Bruce (one of the inhibitors of apoptosis proteins) (55, 56).

### 3.4 Cell proliferation

EBV-encoded miRNAs have been reported to exert dual effects on cell proliferation in EBVaGC. Meng-He Zhao et al. found that, in EBVaGC cells, miR-BART2-5p and miR-BART11-5p were overexpressed and promoted cell proliferation by down-regulating *RB* and *p21* expression (44). In addition, miR-BART19-3p induced cell proliferation by regulating the expression of Wnt inhibitory factor 1 (*WIF1*), Nemo-like kinase (*NLK*), and *APC* (57). Conversely, Dan Wang and colleagues reported that miR-BART6-3p/LOC553103/stathmin 1(STMN1) axis regulated the expression of cell cycle-associated proteins: cycle-associated proteins p27, Cyclin E1 (CCNE1), Cyclin D1 (CCND1), and cyclin-dependent kinase 4 (CDK4), inhibiting cell proliferation (58). Notably, miR-BART16 has been demonstrated bidirectional impacts in EBVaGC cells (59, 60). In Hooykaas’ work, they showed that miR-BART16 targeted the key transcriptional co-activator cAMP response element-binding protein (CBP) in the IFN signaling transduction, resulting in the downregulation of CBP in the tumor cells and ultimately suppressed the anti-proliferative effect of IFN-α (59). In another study, Zhang and colleagues observed that the EBVnGC cells transfected with miR-BART16 mimics had a reduced growth rate and exhibited a notably increased growth rate while inhibiting the endogenous expression of miR-BART16 (60).

### 3.5 Immune responses

EBV-encoded miRNAs regulate the immune responses in EBVaGC. Chan Jin Yoon et al. indicate that transfection of miR-BART5-5p in EBVnGC cell lines directly targets the protein inhibitor of activated signal transducer and activator of transcription 3 (PIAS3), leading to PIAS3/activated signal transducer and activator of transcription 3 (pSTAT3) -dependent up-regulation of PD-L1 (61). Jie Wang et al. also demonstrated that miR-BART11 and miR-BART17-3p promoted the expression of PD-L1 by targeting *Forkhead Box P1 (FOXP1)* and *Polybromo-1* (*PBRM1)* gene in ex vivo assays (62). Additionally, miR-BART3-3p was shown to inhibit the infiltration of NK cells and macrophages into EBVaGC by altering the senescence-associated secretory phenotype (SASP) (63).

### 3.6 Autophagy

EBV-encoded miRNAs have been implicated in the promotion of malignant transformation of cells, potentially *via* their involvement in autophagy. Unc-51-like kinase 1 (ULK1) is a serine/threonine protein kinase that plays an important role in autophagy initiation (36). According to Duo Shi et al., luciferase reporter assay revealed that miR-BART2-3p directly targets ULK1, leading to decreased expression of autophagy-related proteins.

### 3.7 Section summary

EBV-encoded miRNAs are considered to be essential participants in the carcinogenesis of EBVaGC. They are involved in diverse cellular processes, forming an extensive and complex functional network. However, our current understanding of their roles in EBVaGC remains limited, highlighting the need to address several knowledge gaps.

Firstly, particular miRNAs target multiple genes, while multiple miRNAs, such as miR-BART2-5p and miR-BART11-5p, target specific genes. This intricate interplay underscores the importance of fully elucidating the functional roles of EBV-encoded miRNAs in EBVaGC. Secondly, certain miRNAs exhibit bidirectional effects, for instance, miR-BART12 and miR-BART16, which have opposite impacts (suppression/promotion) on migration and cell proliferation regarding their targeted loci. These bidirectional effects pose challenges to their further application and necessitate deeper investigation. Thirdly, most of the presented evidence is derived from *in vitro* studies using EBVaGC and EBVnGC cell lines, rather than clinical tumor tissues. Although *in vitro* studies have indicated that miRNAs regulate immune responses and potentially modulate the immune microenvironment, comprehensive understanding and validation of these mechanisms require *in vivo* investigations. Furthermore, the lack of standardized and rigorous study design, including the inclusion of EBVnGC samples and adjacent normal tissue as controls, as well as limited data from animal models, hampers comprehensive analysis.

## 4 Prognostic impact of the miRNA

Recent clinical data suggested that EBVaGC has a relatively better prognosis than EBVnGC. MiRNAs have been proposed as potential prognostic biomarkers in EBVaGC (64). In an international pooled analysis of 4 599 GC patients from Asia, Europe, and Latin America, EBVaGC was found to be associated with lower mortality (hazard ratio = 0.72; 95% confidence interval 0.61 - 0.86) (65). Additionally, data from a cohort study with 384 GC patients derived from The cancer genome atlas (TCGA) database showed longer relapse-free survival (RFS) and overall survival (OS) for EBVaGC compared to the microsatellite instability (MSI), genomically stable (GS), and chromosomal instability (CIN) subtypes (66).

A retrospective study analyzed clinicopathological information for 847 GC patients (38). Of these patients, 71 were diagnosed with EBV infection. Statistical analysis revealed that the high expression of miR-BART10-3p was associated with lymph node metastasis (P = 0.010), increased expression of miR-BART22 was associated with T stage (P = 0.036), and lymph node metastasis (P = 0.005) (38). Worse OS was observed in EBVaGC patients with high expression of bar10-3p and bar22. Byung Woog Kang et al. examined tumor tissues of 59 EBVaGC patients, compared with 39 normal mucosal tissues, and analyzed miRNA expression by qRT-PCR (12). They reported that 3-year RFS and OS were 76.4% and 78.7%, respectively, with median follow-up of 24.1 months (2.8 - 48.0 months) (12). In the multivariate analyses adjusted for age and clinical stage, high expression of miR-BART20-5p appeared to be associated with shorter RFS (P = 0.034, HR = 6.951, 95% CI = 1.158 - 41.737) (12).

Preliminary investigations exploring miRNAs as diagnostic and prognostic markers have yielded promising results in various tumor types, including colorectal, lung, and ovarian cancer (67–69). However, their clinical effectiveness remains to be further investigated. Several challenges hinder their clinical application, including the limited specificity and sensitivity of miRNA as biomarkers, the absence of standardized and quantitative techniques, and the high detection cost (70). Therefore, it is essential to draw from these experiences and develop miRNA detection methods that are highly sensitive, specific and economically viable for their application in clinical settings.

## 5 Potential applications of miRNA

The treatment of GC commonly involves the use of chemotherapeutic agents such as docetaxel, fluorouracil, etoposide, and irinotecan (71, 72). Related studies have demonstrated that miR-BART15-3p increases chemosensitivity to 5-fluorouracil (5-FU) (55), while miR-BART20-5p, miR-BART5-3p, and miR-BART3-3p respectively confer resistance to 5-FU, docetaxel, etoposide, and irinotecan (63, 72, 73). Radiotherapy significantly induced p53 expression and apoptosis, while miR-BART5-3p can inhibit the expressions of PARP, p53 and p21 induced by 4Gy ionizing irradiation (72). These findings suggested that miR-BART5-3p contributed to chemotherapeutic drug resistance in EBVaGC cells and the radiotherapy-induced degradation of p53 protein. In the preceding section, we awarded that miR-BART5-5p, EBV-miR-BART11, and EBV-miR-BART17-3p can upregulate PD1 expression. These phenomena have prompted us to hypothesize that miRNA may be involved in immune regulation, thereby affecting the efficacy of immune therapy.

MiRNAs are a class of molecules that have emerged as promising targets for cancer therapy, with an increasing number of their roles being revealed in numerous studies annually (74–76). The advantages of miRNAs as a therapeutic approach include their small size, sufficient stability, easy chemical modification, lower immunogenicity, and ability to target and bind to multiple target mRNAs (77). However, the clinical application of miRNAs in other types of tumors has encountered difficulties (78). Public information statistics showed that only 11 miRNA drugs had entered clinical trials, with most still in clinical development, and none have advanced to Phase III. This stagnation is also true for EBVaGC, most likely due to the extensive and complex effects of miRNA, which can bind to multiple target mRNAs and form a vast and intricate regulatory network through upregulating or downregulating the expression levels of target miRNA. MiRNAs exhibit a dual effect on enhancing or inhibiting tumor progression, and there is also cross-talk between different information molecules and signaling pathways, forming a complex regulatory network. To increase our understanding of the roles and mechanisms of miRNAs in the oncogenesis and development of EBVaGC, we should draw on the experiences of other tumor types and explore new approaches. These include searching for relevant miRNAs and introducing miRNA or miRNA antagonists into patients to regulate downstream gene expression and ultimately achieve therapeutic effects.

## 6 Conclusion

Current evidence suggests that EBV-encoded miRNAs play a crucial role in the carcinogenesis of EBVaGC by being involved in various cellular processes, such as migration, cell cycle, apoptosis, cell proliferation, immune response, and autophagy. While ongoing investigations of miRNA functions and underlying mechanisms are underway, preliminary explorations of their clinical applications have also been initiated. However, before miRNAs can be utilized in clinical settings, several significant challenges must be addressed. Firstly, the understanding of the integrated role of miRNAs in EBVaGC is limited due to the need for studies focusing on clinical tissue samples and animal models with adequate sample sizes, which leads to incomplete knowledge of its complex regulatory network. Secondly, the low reproducibility of research results is also a significant challenge, as findings from different studies cannot be validated. Thirdly, the lack of miRNA detection methods with high sensitivity and specificity, and low costs, prevents the standardization of quantitative EBV-miRNA data, resulting in difficulty in providing clinically meaningful information. Last but not least, miRNAs are involved in various cellular processes and constitute an extensive, complex functional network, making it difficult to achieve specific gene silencing, leading to unforeseeable side effects, which is the main reason for the failure of clinical trials to date (79, 80).

Animal and *in vitro* cell culture models are badly-needed to investigate miRNA function and develop therapeutic interventions. Standardized, economical, and unified detection criteria are prerequisites for clinical application. After comprehensive recognition of the oncogenic mechanism, miRNA can be further applied clinically. Despite the challenges presented, potential avenues for the development of clinical applications may be explored through the use of synthetic miRNAs (miRNA mimics), recombinant expression vectors encoding miRNA sequences, and oligonucleotide-based miRNA inhibitors (anti-miRNAs) (78).

The exploratory work has achieved preliminary results, and some EBV miRNAs have been shown to be useful as diagnostic, prognostic, and therapeutic biomarkers for EBVaGC in *in vitro* experiments. With the discovery of new target genes, the role of EBV miRNA will be further uncovered, and the development of EBV miRNA-targeted therapy will undoubtedly bring a new revolution in the treatment of EBVaGC in the future.

## Author contributions

TL and TH: conceptualized and wrote the manuscript. YM and TH: guidelines and proofreading. All authors contributed to the article and approved the submitted version.
